# Toxicological Features of *Catha edulis* (Khat) on Livers and Kidneys of Male and Female Sprague-Dawley Rats: A Subchronic Study

**DOI:** 10.1155/2012/829401

**Published:** 2012-11-27

**Authors:** Abdulsamad Alsalahi, Mahmood Ameen Abdulla, Mohammed Al-Mamary, Mohamed Ibrahim Noordin, Siddig Ibrahim Abdelwahab, Aied M. Alabsi, Abdrabuh Shwter, Mohammed A. Alshawsh

**Affiliations:** ^1^Department of Molecular Medicine, Faculty of Medicine, University of Malaya, 50603 Kuala Lumpur, Malaysia; ^2^Department of Organic Chemistry, Faculty of Pharmacy, Sana'a University, Sana'a, Yemen; ^3^Department of Pharmacy, Faculty of Medicine, University of Malaya, 50603 Kuala Lumpur, Malaysia; ^4^Faculty of Dentistry, University of Malaya, 50603 Kuala Lumpur, Malaysia

## Abstract

Hepato- and nephrotoxicity of Khat consumption (*Catha edulis* Forskal) have been evoked. Therefore, this study was conducted to evaluate such possible hepatorenal toxicity in female and male Sprague-Dawley rats (SD rats) focusing primarily on liver and kidney. In addition, female and male rats were investigated separately. Accordingly, forty-eight SD-rats (100–120 g) were distributed randomly into four groups of males and female (*n* = 12). Normal controls (NCs) received distilled water, whereas test groups received 500 mg/kg (low dose (LD)), 1000 mg/kg (medium dose (MD)), or 2000 mg/kg (high dose (HD)) of crude extract of *Catha edulis* orally for 4 weeks. Then, physical, biochemical, hematological, and histological parameters were analyzed. Results in Khat-fed rats showed hepatic enlargement, abnormal findings in serum aspartate aminotransferase (AST), and alkaline phosphatase (ALP) of male and female SD-rats and serum albumin (A) and serum creatinine (Cr) of female as compared to controls. In addition, histopathological abnormalities confirmed hepatic and renal toxicities of Khat that were related to heavy Khat consumption. In summary, Khat could be associated with hepatic hypertrophy and hepatotoxicity in male and female SD-rats and nephrotoxicity only in female SD-rats.

## 1. Introduction

Khat is the most common name for *Catha edulis *plant [1] which is consumed for its psychostimulatory effect [2]. Unfortunately, Khat become a serious public health problem in Yemen [2]. The chewing of Khat leaves has involved at least 80% of adult males [3] and extended to women, too [4]. The WHO (2003, 2006) reported that Khat consumption has become a common problem that affects the health aspects of life [4]. In fact, many adverse effects have been associated with Khat consumption [2]. Accordingly, prolonged exposure to Khat could result in psychoneurological disturbances such as neurosis [5]. In addition, increased diastolic blood pressure [6] and vasoconstriction of coronary vasculature were also reported [7]. More commonly, gastritis [8], hemorrhoids, and duodenal ulcer had a higher prevalence among Khat chewers [9]. Furthermore, Luqman and Danowski reported that liver cirrhosis that was observed among Yemeni Khat chewers might be due to Khat consumption, but at that time it was not further investigated [10], and hepatotoxicity of Khat chewing is still debated in humans [11–13]. In animals, the administration of crude extract of Khat to New Zealander white rabbits for three months suggested toxic hepatocellular jaundice as well as histopathological abnormalities in livers of such animals [14]. Likewise, a companion study on the same species of animals with the same dose levels of crude extract for six months supported the former three-month findings; however, after six months histopathological evidence from liver sections suggested periportal fibrosis as an initial indicator of liver cirrhosis without apparent damage to kidneys [15]. As a limitation, those studies did not include females for the hepatotoxicity studies and evaluation of Khat nephrotoxic effects in either males or females was only minimally investigated. In addition, the doses of crude extract that were given to animals were selected to be in the average of 150–200 g of fresh Khat leaves according to Kalix [16], which was less than that suggested by Al-Habori et al. [15]. However, there are no comparative studies about the effects of the different dose levels of Khat on human in terms of body weight. Recently, several reports were published on severe liver diseases in Khat users [11, 17, 18].

Our study was carried out to predict whether oral administration of crude extract of Khat for 4 weeks could be associated with hepatorenal toxicity in either male or female Sprague-Dawley rats. In addition, female SD-rats and actual dose levels were included in the scope of the current study. Moreover, several parameters were selected to obtain a clearer image of Khat toxicity signature on liver and kidney including physical, serum biochemical, hematological, and especially histopathological parameters.

## 2. Methodology

### 2.1. Materials

#### 2.1.1. Plant Material

Four kilograms of fresh material of Hamadani-type of Khat (the same type that chewers purchase and consume) were purchased from a local Yemeni market, Sana'a, Yemen. The fresh materials of Khat were wrapped in a plastic bundle just as chewers do. Next, the fresh materials of Khat were transferred to the Pharmacognosy Laboratory, Faculty of pharmacy, Sana'a University to be verified. Voucher specimens were kept in Pharmacognosy Department as a reference. The fresh materials were washed to remove dust and debris with distilled water. The edible parts were cut off to get three kilograms of fresh material and subjected to air-drying for two weeks in a dark place. After 2 weeks, the dried materials were ground in a heavy duty blender (Moulinex-Trade blender). Finally, a net weight equal to 500 g of the dried ground material was obtained.

#### 2.1.2. Sample Extraction

The dried material was extracted with sufficient quantities of 80% methanol (Sigma-Aldrich and Fisher) using a Buchi-type soxhlet apparatus (5 mL of solvent per 1 g of dry material). Then, the resulted extract was evaporated using a Buchi-type rotary evaporator under vacuum at 40°C until dryness. Finally, the resulted yield (100 g dried extract) was kept in a desiccator until the time of use.

#### 2.1.3. Experimental Animals

Forty-eight healthy male and female Sprague-Dawley rats (100–120 g) were obtained from the Animal House Unit, Faculty of Medicine, University of Malaya, Malaysia.

### 2.2. Methods

#### 2.2.1. Study Design

The study was conducted under ethic number of (PM/07/05/2008/1111/MAA (a) (R)) that has been approved by the Ethic Committee, Animal House Unit, University of Malaya. The procedure followed the instructions of Good Laboratory Practice (GLP) [19] and guidelines of Organization of Economic Co-operation and Development (OECD); testing of chemicals 407 [20] Rats were allowed to acclimatize the working conditions one week prior to treatment, kept in a separated cage (1 rat per cage) under standard conditions of artificial light (12 hr light 12 hrs dark), temperature (25 ± 3°C), and relative humidity (60% ± 10%), and fed on a standard diet and water ad libitum. By the end, SD-rats were transferred into wire mesh-bottomed cages (to avoid coprophagy) with free access to water, but the food was withheld overnight to collect fasting blood samples via cardiac puncture for serum biochemical assay and blood film. All rats were sacrificed 24 hours after the last treatment. On the day of sacrificing, rats were anaesthetized intramuscularly with ketamine. Rats were killed through increasing ketamine dose. Then, rats were dissected to collect livers and kidneys.

#### 2.2.2. Grouping and Dosing of Rats

Rats were randomly selected and distributed into two main groups of males and females (*n* = 24), which were allocated into four groups (*n* = 6) designated as high dose (HD), medium dose (MD), low dose (LD), and normal control (NC). Rats were given a single daily dose of crude extract of Khat suspended in pure distilled water according to the body weight of each rat by oral gavage. The dose levels were selected and calculated typically as the daily consumed amounts of fresh leaves by Khat chewers [8]. The dosages of Khat designated to be administered to rats were 2000 mg/kg as high dose (HD), 1000 mg/kg as medium dose (MD), 500 mg/kg as low dose (LD), and 10 mL/kg of distilled water for normal control (NC).

#### 2.2.3. Clinical Observation of Rats

Prior to the start of the study, all rats were clinically observed for any abnormal behavior, physical abnormalities, mortality, and morbidity at least once daily.

#### 2.2.4. Physical Parameters

The body weight of rats was taken once weekly, at the first day of treatment and the day of sacrificing. Livers were washed in normal saline and blotted with filter paper. The weight of livers was taken to obtain absolute liver weight for each rat. By dividing liver weight on body weight, relative liver weight was obtained for each rat.

#### 2.2.5. Serum Biochemical Parameters

Fasting blood samples were collected into plain tubes. Then, tubes were allowed to stand to give full clotting. Then, tubes were centrifuged immediately at 2500 ×g for 15 minutes to obtain a clear supernatant. Then, 48 serum-containing tubes were admitted to the Laboratory Department, Faculty of Medicine, University of Malaya to carry out the biochemical parameter analysis. Serum biochemical parameters included alkaline phosphatase (ALP), alanine aminotransferase (ALT), aspartate aminotransferase (AST), creatine kinase (CK), amylase (AM), gamma-Glutamyl transpeptidase (GGT), total bilirubin (TB), albumin (A), creatinine (Cr), urea (BUN), sodium (Na), potassium (K), chloride (Cl), and carbon dioxide (CO_2_).

#### 2.2.6. Hematological Parameters

The other part of blood was collected into 48 EDTA-containing tubes. Then, those blood samples were admitted to the Laboratory Department, Faculty of Medicine, University of Malaya. Blood samples were examined for various parameters including hemoglobin (HB), hematocrit (HCT), red blood cells (RBC), mean corpuscular volume (MCV), mean corpuscular hemoglobin (MCH), mean corpuscular hemoglobin concentration (MCHC), White blood cells (WBC), and platelets (PLT).

#### 2.2.7. Histopathology Study

After autopsy, livers and kidneys were washed in normal saline, blotted with filter paper, and visualized for any surface visible lesions immediately after harvesting and weighed. Then, organs were put in neutral phosphate buffer solution. After that, biopsies were obtained, fixed in 10% neutral formalin, processed, dehydrated, and embedded in paraffin wax. The 5 *μ*m sections in thickness were stained according to the usual hematoxylin-eosin procedure to be microscopically examined (20x magnification).

#### 2.2.8. Statistical Analysis

The results were computed statistically with SPSS software package version 17 using one-way analysis of variance (ANOVA) for mean difference between groups, and males and females were analyzed separately (*n* = 6) and *P* value was set on *P* < 0.05. The values were expressed as mean ± SEM. Then post hoc Dunnett test (two-sided) was followed for comparing the tested groups to the single normal control at *P* < 0.05 and *P* ≤ 0.01. Otherwise, nonparametric tests were employed when the data were not normally distributed so that nonparametric Kruskal Wallis test was used to compare median difference between groups which was followed by Mann-Whitney test to compare each Khat treated group to the normal control group.

## 3. Results

All rats were in good health and neither mortality nor morbidity was observed.

### 3.1. Physical Parameters

Body weight (BW) of male SD-rats was significantly (ANOVA *P* < 0.05) different between groups, and post hoc Dunnet test indicated that mean body weight of the male HD-group was significantly (*P* < 0.05) lower than that of male NC-group (by 19%). In addition, absolute liver weight (ALW) of males was nonsignificantly (ANOVA, *P* ≥ 0.05) different between groups. However, relative liver weight (RLW) of male SD-rats was significantly (ANOVA, *P* < 0.05) different between groups and post hoc Dunnet test indicated that mean value of the male HD-group was significantly (*P* < 0.05) greater than that of male NC-group (by 21%) see Table 1.

BW of females were nonsignificantly (ANOVA *P* ≥ 0.05) different between NC, LD, MD, and HD. However, absolute liver weight (ALW) of female SD-rats was significantly (ANOVA, *P* < 0.05) different between groups and post hoc Dunnet test indicated that mean value of HD was greater than that of female NC-group (*P* ≤ 0.01) (by 34%). Likewise, relative liver weight (RLW) of female SD-rats was significantly (ANOVA, *P* < 0.05) different between groups and post hoc Dunnet test indicated that mean values of HD, MD, and LD were significantly (*P* ≤ 0.01) greater than that of female NC (by 56%, 28%, and 25%, resp.) see Table 1.

### 3.2. Serum Biochemistry

#### 3.2.1. Liver Function Test

Serum activity of alkaline phosphatase (ALP) in male SD-rats was significantly (ANOVA, *P* < 0.05) different between the groups. Accordingly, post hoc Dunnet test indicated that mean ALP of HD-group was significantly (*P* < 0.05) higher than that of NC (by 45%). Serum activity of alanine aminotransferase (ALT) of male SD-rats seemed significantly different between groups (ANOVA, *P* < 0.05); however, post hoc Dunnet test indicated a non-significant difference (*P* ≥ 0.05) of Khat groups compared to that of NC. Unlike ALT, serum activity of aspartate aminotransferase (AST) in male SD-rats was significantly (ANOVA, *P* < 0.05) different between groups. Post hoc Dunnet test indicated that mean AST value of HD was significantly (*P* < 0.05) greater than that of NC (by 34%). In addition, serum total bilirubin (TB) of male SD-rats was significantly (*P* < 0.05) different as indicated by Kruskal-Wallis test; Mann-Whitney test indicated that HD and MD were significantly greater than that of NC (see Table 2). Conversely, serum albumin (A) of male SD-rats was nonsignificantly (ANOVA, *P* ≥ 0.05) different from that of NC (see Table 2). Moreover, serum activities of gamma-glutamyl transpeptidase (GGT) and serum levels of unconjugated bilirubin of male SD-rats were undetectable in both the tested groups and NC.

Serum activity of ALP of female SD-rats was significantly (ANOVA, *P* < 0.05) different between groups. Post hoc Dunnet test indicated that the mean values of HD and MD were significantly (*P* < 0.05) greater than that of NC (by 57% and 48%, resp.). Serum activity of alanine aminotransferase (ALT) of female SD-rats seemed significantly (ANOVA, *P* < 0.05) different between groups; however, mean values of HD, MD, and LD were not different from NC (*P* ≥ 0.05). Serum activity of aspartate aminotransferase (AST) of female SD-rats was significantly (ANOVA, *P* < 0.05) different between groups, and post hoc Dunnet test indicated that mean values of HD and MD were significantly (*P* ≤ 0.01) greater than that of NC (by 34% and 28%, resp.). In addition, the serum total bilirubin (TB) of female SD-rats was nonsignificantly (*P* ≥ 0.05) different from NC as indicated by Kruskal-Wallis test. Conversely, the serum albumin (A) of female SD-rats was significantly (ANOVA, *P* < 0.05) different between groups and post hoc Dunnet indicated that mean values of both HD- and LD were significantly (*P* ≤ 0.01) lower than that of NC (by 20%). Moreover, serum activities of gamma-glutamyl transpeptidase (GGT) and serum levels of unconjugated bilirubin in female SD-rats were undetectable in both the tested groups and NC see Table 2.

#### 3.2.2. Serum Enzymes

No significant (ANOVA, *P* ≥ 0.05) difference was demonstrated in serum activities of creatine kinase (CK) and amylase (AM) in both male and female SD-rats see Table 3.

#### 3.2.3. Renal Biomarkers

Serum creatinine (Cr) in male SD-rats was significantly (ANOVA, *P* < 0.05) different between groups. However, post hoc Dunnet test indicated that the mean values of HD, MD, and LD were significantly (*P* < 0.05) lower than that of NC. Likewise, serum urea (BUN) of male SD-rats was significantly (ANOVA, *P* < 0.05) different between groups; however, post hoc Dunnet test indicated that mean value of MD was significantly (*P* < 0.05) lower than that of NC (by 30%). In Addition, the serum levels of sodium (Na), potassium (K), chloride (Cl), and carbon dioxide (CO_2_) were nonsignificantly (ANOVA, *P* ≥ 0.05) different between groups see Table 4.

Serum creatinine of female SD-rats was significantly (ANOVA, *P* < 0.05) different between groups. Post hoc Dunnet test indicated that mean values of MD and LD were greater than that of NC (by 27% and 28%, resp.). On the other hand, serum urea (BUN) in female SD-rats was nonsignificantly (ANOVA, *P* ≥ 0.05) different between groups. Like BUN, the serum levels of sodium (Na), potassium (K), chloride (Cl), and carbon dioxide (CO_2_) of female SD-rats were nonsignificantly (ANOVA, *P* ≥ 0.05) different between groups see Table 5.

### 3.3. Hematological Study

Red blood cells (RBC), white blood cells (WBC), platelets (PLT), hemoglobin (HGB), hematocrit (HCT), and mean corpuscular hemoglobin (MCH) values in blood film of male SD-rats were nonsignificantly (ANOVA, *P* ≥ 0.05) different between groups. Conversely, the mean corpuscular hemoglobin concentration (MCHC) in male SD-rats was significantly (ANOVA, *P* < 0.05) different between groups, and post hoc Dunnett test indicated that mean values of MD and LD were significantly (*P* < 0.05) greater than that of NC (by 2% each) see Table 6.

Red blood cells (RBC), platelets (PLT), hemoglobin (HGB), and hematocrit (HCT) in blood film of female SD-rats were nonsignificantly (ANOVA, *P* ≥ 0.05) different between groups. Conversely, the value of white blood cells (WBC) of female SD-rats was significantly (ANOVA, *P* < 0.05) different, and post hoc Dunnet test indicated that mean value of HD was significantly (*P* < 0.05) higher than that of NC (by 38%). In addition, the mean corpuscular volume (MCV) of female SD-rats was significantly (ANOVA, *P* < 0.05) different between groups, and post hoc Dunnet test indicated that HD was significantly (*P* ≤ 0.01) greater than NC (by 2%). In addition, the mean corpuscular hemoglobin (MCH) in female SD-rats was significantly (ANOVA, *P* < 0.05) different between groups, and post hoc Dunnet test indicated that the mean MCH values of HD (*P* < 0.01), MD (*P* ≤ 0.01), and LD (*P* < 0.05) were significantly higher than that of NC (by 8%, 6% and 4%, resp.). Moreover, mean corpuscular hemoglobin concentration (MCHC) in female SD-rats was significantly (ANOVA, *P* < 0.05) different between groups and post hoc Dunnet test indicated that the mean MCHC values of both HD and MD were significantly (*P* ≤ 0.01) greater than that of NC (by 4%) see Table 7.

### 3.4. Histopathology Examination

**Figure 1:**
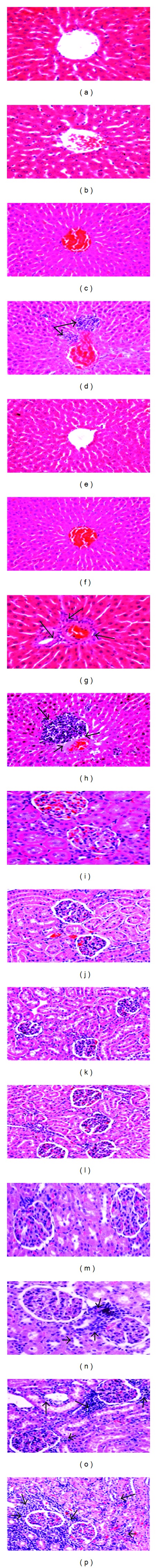
Effect of Khat on histological sections of liver and kidney of male and female rats. (a) Normal control of males with normal liver parenchyma, (b) low dose of males with normal liver parenchyma, (c) medium dose of males with normal liver parenchyma, (d) high dose of males with moderate necrotic lesions around the central vein, (e) normal control of females with normal liver parenchyma, (f) low dose of females with normal liver parenchyma, (g) medium dose of females with slight necrotic lesion around the central hepatic vein, (h) high dose of females with a moderate necrotic lesion around the central hepatic vein, (i) normal control of males with normal renal parenchyma, (j) low dose of males with normal renal parenchyma, (k) medium dose of males with normal renal parenchyma, (l) high dose of males with normal renal parenchyma, (m) normal control of females with normal renal parenchyma, (n) low dose of females with mild necrotic lesion in the renal parenchyma, (o) medium dose of females with moderate necrotic lesion in the renal parenchyma, (p) high dose of females with severe necrotic lesions in the renal parenchyma (H & E stain 20x).

Microscopic examination of liver sections of normal controls of both male and female SD-rats showed uniform hepatocytes, intact cytoplasm, prominent nuclei of cells, and uncongested central vein. In addition, no necrotic lesions, fatty changes, or inflammatory signs were observed in those animals. Similarly, liver sections of LD- and MD-groups of male and those of female SD-rats in LD-groups showed normal hepatocytes architecture, preserved-cytoplasm without any apparent necrotic lesions. However, liver sections of HD-groups of khat-treated male and female SD-rats showed a degenerative vacuolation and coagulative necrosis in zone 3 (pericentral region) and degenerative changes in persisting parenchyma with congestion and hemorrhage. There were dilatation of sinusoids and mononuclear inflammatory infiltrates and Kupffer cells around the central vein and portal tracts. Such changes were minimally apparent in liver sections of MD-group of female SD-rats (Figure 1).

Microscopic examination of renal sections of normal control groups of male and female SD-rats demonstrated typical and normal histological features of tubules, Malpighian corpuscles, glomerular capillaries, and Bowman's capsule. Similarly, renal sections of male SD-rats that were treated with LD, MD, and HD of crude extract of Khat showed normal cortical tubules, and no abnormal histological changes were observed in Malpighian corpuscles, glomerular capillaries, or Bowman's capsule. However, renal sections of female SD-rats that received LD, MD, and HD of crude extract of Khat showed different degrees of histopathological changes according to the dose level. Such changes were characterized by atypical tubules, amorphous Malpighian corpuscles, and invasive infiltrative inflammatory cells. The glomerular capillaries in Malpighian corpuscles were hypertrophied, some of them were destructed, and Bowman's capsules seemed dilated (Figure 1).

## 4. Discussion

Neither morbidity nor mortality was observed. However, the reducing effect of Khat on the body weight of male SD-rats with high dose (HD) could be a primary indicator of anorexigenic effect [21, 22] and might result from the local astringent effects of Khat on intestinal absorption [23]. In addition, the higher the dose level, the greater the reduction in body weight [24]. It was found that the decrease in body weight was correlated to a decrease in leptin level. Therefore, rats that were treated with Khat showed a significant decrease in body weight and level of leptin [25]. However, the effect of Khat on leptin has been still questionable in female rats since the study was not elaborated to consider gender. On the other hand, the growth retardation of animals treated with Khat extract might be attributed to the presence of tannins and other polyphenolic compounds, since tannins and polyphenolic compounds could inhibit the digestive enzymes [26] and consequently, the nutrients absorption would be impaired. In human, the effect of khat on the weight of chewers has still been controversial particularly with the absence of well-documented studies about such interaction and the association of chewing with smoking. In addition, it was found that women were less involved in Khat chewing than men [27]. Accordingly, it could be expected that men may lose weight more than women, since smoking was associated with a decrease in leptin expression [28]. However pregnant women, who were Khat chewers during pregnancy, give birth to underweight newborns [29]. In our study, the interpenetration of the observed reduction in body weight of male rather than female SD-rats was still not understood, and needs further investigation. Anyway, our findings were consistent with the results of other researchers [23–25].

The observed hepatic enlargement that male and female SD-rats developed seems to be due to exposure to Khat. Hepatic enlargement represents a feature of liver regeneration process which is experienced clinically after liver injury. A lot of xenobiotics were associated with hepatic enlargement due to a direct effect of xenobiotics on the size of hepatocytes [30] or an inflammatory response [31] that were associated with the histopathological changes in hepatocytes of rats. Such changes were characterized by mononuclear infiltrates, dilatation of the sinusoids, vacuolar and coagulative necrosis, and the congestive and hemorrhagic degenerative changes in hepatocytes parenchyma particularly in livers of the HD-group of male SD-rats as well as HD-and MD-dose of female SD-rats. On the other hand, it could be suggested that the association of hepatic enlargement with histopathological changes could be supported by the similar degrees of severity of hepatic enlargement and the histopathological changes so that the higher degree of histopathological changes, the greater the hepatic enlargement. The degenerative vacuolar lesions in liver probably impaired the liver function of rats and orally administered khat had a direct cytotoxic effect on liver cells.

The observed elevation in serum activity of alkaline phosphatase (ALP) in the HD-group of male SD-rats and HD- and MD-groups of female SD-rats indicates a possible hepatotoxicity [32]. The elevation of several markers would be of a greater diagnostic value than a single one [33]. The lack of specificity of AST, ALP, and GGT cannot authorize any conclusion because such enzymes are ubiquitous in various tissues and serum elevation could represent not only liver, but also heart, bones, and muscles damages [34]. Moreover, the inhibited activity of serum alanine aminotransferase (ALT) could indicate normal hepatic function because of its specificity as a marker for hepatocytes integrity [34–38]. Another possibility could be the establishment of a covalent binding between certain components of Khat and ALT [33]. Although serum ALT was reported to be a poor marker in predicting hepatotoxicity in rats [39], the higher serum activity of aspartate aminotransferase in HD-group of males as well as HD- and MD-groups of females than that of normal control rats (NC) suggests leakage into circulation from ruptured cell membranes of hepatocytes upon exposing to injury [34]. Although AST is widely distributed in various organs, its concentration in hepatocytes is still the highest [35]. Pancreatic damage was unlikely, since serum amylase (AM) was nonsignificantly changed despite its lack of specificity [40]. Again, elevations of AST due to straited muscles injury should be associated with parrallel elevations in serum creatine kinase (CK) as a marker of skeletal [34, 35, 41] and/or heart muscles injury [24]. However, CK in the tested groups of male and female SD-rats was nonsignificantly different from that of NC. Histopathological examination of biopsies from heart muscles indicated no abnormal findings in both male and female SD-rats. Elevations of GGT in rodents were rarely detected, even if liver was exposed to a known hepatotoxic compound, so this does not exclude hepatotoxicity of khat [33]. Consequently, elevations of serum AST were of hepatic origin rather than cardiac, muscular, or pancreatic origin. In addition, the low levels of serum albumin (A) of female SD-rats with HD and LD suggested a defect in the hepatic synthesis capacity [38, 42]. It is known that albumin synthesis is restricted to liver [42]. Accordingly, our findings were consistent with those reported by Al-Mamary et al. (2002) with Khat exposure for 3 months and with Al-Habori et al. (2002) with Khat exposure for 6 months [15, 43]. However, the elevation of serum activity of ALT in the rabbits in the aforementioned article was contrasting to the findings of our study because ALT was higher in rabbits which maybe because the doses given to rabbits were higher and exposure was longer than that in the current study. So, there is too much uncertainty on biological data to draw back any conclusion and only histological findings assess Khat hepatotoxicity in rats.

Similarly, serum creatinine (Cr) and urea (BUN) could indicate the effect of xenobiotics on kidney structure and function [31, 33]. But the findings of normal activity of Cr and BUN and serum electrolytes in males indicated normal renal function. Nonetheless, elevated serum values of Cr in females particularly in the MD- and LD-Khat groups indicated impaired kidney function or deshydratation (dehydration). Accordingly, our findings were consistent with those results of Al-Mamary et al. [14] with Khat exposure but contrasting to Al-Habori et al. [15] who suggested normal kidney functions in animals after six-month exposure to Khat. The hematology profile was found to be normal except for a mild elevation in white blood cells (WBC) in the HD-group of females. So, despite physical, biochemical, and hematological findings only histological examination of liver and kidney biopsies fit with Khat hepato- and nephrotoxicity. Actually, Khat had direct cytotoxic effects on liver and kidney which were confirmed by the microscopic examination of hepatic and renal sections. Such changes in liver parenchyma were demonstrated in the form of coagulative necrosis (in zone 3) with hemorrhage, mononuclear infiltrates of inflammatory cells, and vacuolar degeneration in HD-group of males as well as MD- and HD-groups of female SD-rats, which indicated a regeneration process of liver [44] because of exposure to khat. Examination of histopathological changes of renal sections indicated that only kidneys of female SD-rats were affected with Khat in different degrees of severity according to dose so that the higher the dose, the greater the destructive changes in kidney structures. Such changes were characterized by invasive infiltrates of inflammatory cells, atypical tubules, and amorphous Malpighian corpuscles; hypertrophied and destructed capillaries of Malpighian corpuscles were hypertrophied and dilated Bowman's capsules. Histopathological study on liver and kidney was consistent with those changes reported by Al-Mamary et al. [43] as well as it was consistent with the hepatic histopathological study conducted by Al-Habori et al. [15], but no periportal fibrotic changes were observed, which may be due to the longer duration of Khat administration to rabbits for six months.

The findings of study suggest the elaboration of the study to clarify the possible effects of Khat, leptin, and smoking on weight of male and female animals and khat chewers. In addition, the observed hepatic hypertrophy needs further investigation, since it sometimes may be due to physical congestion or hyperplasia. Reconsidering the assessment of the specificity of hepatic biomarkers in disclosing hepatotoxicity of khat is required, and the employment of tissue homogenate, molecular techniques, and immunohistochemistry may be more informative.

Up to now, it has been not reported that the toxic effects exerted by Khat on livers and kidneys of rats can be related either to the content of Khat leaves [10] or to the pesticides [13, 45] that are sprayed on Khat trees to improve harvesting [46]. Accordingly, further study should be performed to clarify such ambiguity through comparing toxicity of pesticide sprayed-Khat to that of pesticides free-Khat. In addition, our study demonstrated that oral administration of Khat to rats was associated with an inhibited activity of the specific hepatic biomarker, alanine aminotransferase (ALT), which could be due to depletion of pyridoxine (vitamin B6) stores [47]. Therefore, the study of the relationship between Khat chewing, serum ALT, and vitamin B6 should be considered.

Our study might provide preliminary evidence of the possible hazards of khat consumption on liver and kidney, so physicians should consider khat as a relevant factor, especially since many researchers reported that khat chewing is associated with hepatotoxicity in human [11, 13, 17, 18].

## 5. Conclusion

Administration of Khat to male and female SD-rats was not safe and resulted in hepatorenal toxicity on short-term exposure assessed by histological lesions. Hepatotoxicity was common in both male and female SD-rats with hepatic hypertrophy, elevated liver enzymes, and zone 3 necrosis on histology. In addition, Khat-induced nephrotoxicity was limited to female and not to male SD-rats. Since hepatorenal toxicity of Khat has now been established both clinically and experimentally, its use should be strongly discouraged by public health authorities.

## Figures and Tables

**Table 1:** Body weight and relative liver weight of male and female SD rats.

Dose (mg/kg)	BW (g)		ALW (g)		RLW (W/W %)	
	Male	Female	Male	Female	Male	Female
NC (10 mL/kg)	200.13 ± 12.30	159.13 ± 6.98	9.57 ± 0.59	5.22 ± 0.28	3.5 ± 0.2	2.67 ± 0.10
LD (500 mg/kg)	168.25 ± 9.54	143.79 ± 5.26	6.80 ± 0.71	5.87 ± 0.29	3.1 ± 0.2	3.35 ± 0.13*
MD (1000 mg/kg)	169.54 ± 9.75	143.25 ± 4.73	7.23 ± 0.75	5.87 ± 0.27	3.2 ± 0.2	3.42 ± 0.16*
HD (2000 mg/kg)	161.42 ± 8.40*	141.08 ± 4.75	8.77 ± 0.64	7.00 ± 0.24*	4.2 ± 0.2*	4.17 ± 0.17*

BW: body weight; ALW: absolute liver weight; RLW: relative liver weight; HD: high dose; MD: medium dose; LD: low dose; NC: normal control. The treated groups received different doses of crude extract of Khat except NC which received distilled water. The values were expressed as mean ± S.E.M. the symbol (*) denoted a significant difference at *P* < 0.05.

**Table 2:** Liver function test of male and female SD rats.

Dose (mg/kg)	ALP (IU/L)		ALT (IU/L)		AST (IU/L)		A (g/L)		TB (uM/L)	
	Male	Female	Male	Female	Male	Female	Male	Female	Male	Female
NC (10 mL/kg)	155.50 ± 6.22	100.83 ± 13.10	35.33 ± 2.51	39.67 ± 2.47	127.67 ± 4.48	135.17 ± 5.33	12.33 ± 1.75	15.67 ± 0.49	1.5	1.0
LD (500 mg/kg)	147.67 ± 22.31	107.67 ± 3.68	28.33 ± 3.44	25.33 ± 2.14	120.00 ± 15.64	127.00 ± 4.62	11.50 ± 2.74	12.50 ± 0.56^b∗^	2.0*	2.5
MD (1000 mg/kg)	146.00 ± 16.42	149.67 ± 18.70*	27.17 ± 3.11	30.50 ± 1.52	118.33 ± 13.44	173.33 ± 10.85*	9.50 ± 2.43	13.67 ± 0.71	2.0*	2.5
HD (2000 mg/kg)	225.67 ± 19.71*	158.67 ± 10.15*	38.17 ± 1.56	31.50 ± 1.5	170.83 ± 6.30*	180.17 ± 10^b∗^	14.00 ± 1.41	12.50 ± 0.72^b∗^	3.0*	3.0

A: albumin; ALP: alkaline phosphatase; AST: aspartate aminotransferase; ALT: alanine aminotransferase; TB: total Bilirubin, HD: high dose; MD: medium dose; LD: low dose; NC: normal control. The treated groups received different doses of crude extract of khat except NC which received distilled water. The values were expressed as mean ± S.E.M. The symbol (*) denoted a significant difference *P* < 0.05, whereas ( ^b∗^) denoted a significant difference at *P* ≤ 0.01.

**Table 3:** Serum enzymes of male and female SD rats.

Dose (mg/kg)	Male SD-rats		Female SD-rats	
	CK (IU/L)	AM (IU/L)	CK (IU/L)	AM (IU/L)
NC (10 mL/kg)	1708.00 ± 100.61	686.00 ± 48.34	1836.33 ± 332.42	524.67 ± 31.09
LD (500 mg/kg)	1564.83 ± 73.67	731.33 ± 28.59	1380.17 ± 108.49	599.67 ± 59.78
MD (1000 mg/kg)	1699.67 ± 76.16	691.17 ± 43.61	1928.33 ± 84.34	441.83 ± 29.15
HD (2000 mg/kg)	2105.33 ± 291.87	636.67 ± 45.66	1919.17 ± 105.68	593.17 ± 64.25

AM: amylase; CK: creatine kinase; HD: high dose; MD: medium dose; LD: low dose; NC: normal control. The treated groups received different doses of crude extract of Khat except NC which received distilled water. The values were expressed as mean ± S.E.M. No significant difference was noticed between groups and normal controls, *P* ≥ 0.05.

**Table 4:** Renal function test of male SD-rats.

Dose (mg/kg)	Cr (uM/L)	BUN (mM/L)	Na (mM/L)	K (mM/L)	Cl (mM/L)	CO_2_ (mM/L)
NC (10 mL/kg)	39.17 ± 2.22	6.05 ± 0.56	143.00 ± 1.53	4.80 ± 0.24	104.67 ± 1.73	22.15 ± 0.41
LD (500 mg/kg)	26.67 ± 2.25*	4.62 ± 0.31	133.67 ± 9.35	4.37 ± 0.35	103.67 ± 6.11	22.07 ± 1.93
MD (1000 mg/kg)	29.17 ± 3.01*	4.25 ± 0.50*	128.17 ± 9.73	4.30 ± 0.23	94.17 ± 6.59	23.50 ± 1.38
HD (2000 mg/kg)	27.17 ± 0.95*	5.63 ± 0.25	142.83 ± 2.44	4.78 ± 0.16	101.17 ± 1.33	24.12 ± 0.78

Cr: creatinine; BUN: urea; Na: Sodium; K: potassium; Cl: chloride; CO_2_: carbon dioxide; HD: high dose; MD: medium dose; LD: low dose; NC: normal control. The treated groups received different doses of crude extract of Khat except NC which received distilled water. The values were expressed as mean ± S.E.M. The superscript (*) indicated a significant difference (*P *< 0.05).

**Table 5:** Renal function test of female SD rats.

Dose (mg/kg)	Cr (uM/L)	BUN (mM/L)	Na (mM/L)	K (mM/L)	Cl (mM/L)	CO_2_ (mM/L)
NC (10 mL/kg)	26.17 ± 0.87	5.42 ± 0.23	137.50 ± 3.92	4.55 ± 0.16	98.83 ± 2.71	23.18 ± 1.53
LD (500 mg/kg)	33.50 ± 1.34^a∗^	3.97 ± 0.11	130.33 ± 2.64	4.63 ± 0.24	90.00 ± 2.73	19.83 ± 1.18
MD (1000 mg/kg)	33.17 ± 2.30^b∗^	4.72 ± 0.88	125.17 ± 9.27	3.98 ± 0.36	93.17 ± 9.46	20.02 ± 1.69
HD (2000 mg/kg)	28.67 ± 1.31	5.27 ± 0.51	126.83 ± 9.39	4.12 ± 0.22	91.17 ± 7.51	20.08 ± 2.06

Cr: creatinine; BUN: urea; Na: Sodium; K: potassium; Cl: chloride; CO_2_: carbon dioxide; HD: high dose; MD: medium dose; LD: low dose; NC: normal control. The treated groups received different doses of crude extract of Khat except NC which received distilled water. The values were expressed as mean ± S.E.M. The superscript (*) indicated a significant difference, whereas (^a^) and (^b^) denoted a significant difference at *P <* 0.05 and *P* ≤ 0.01, respectively.

**Table 6:** Blood film profile of male SD-rats.

Dose (mg/kg)	RBC (×10^12^/L)	WBC (×10^9^/L)	PLT (×10^9^/L)	HGB (g/L)	HCT (L/L)	MCV (fl)	MCH (pg)	MCHC (g/L)
NC (10 mL/kg)	7.80 ± 0.17	7.48 ± 0.46	560.67 ± 97.02	151.67 ± 2.69	7.48 ± 0.46	64.67 ± 0.49	19.50 ± 0.15	303.33 ± 2.03
LD (500 mg/kg)	7.80 ± 0.07	9.98 ± 0.83	757.83 ± 30.83	158.17 ± 1.42	9.98 ± 0.83	64.67 ± 1.28	20.32 ± 0.25	312.17 ± 2.56*
MD (1000 mg/kg)	7.59 ± 0.06	8.48 ± 0.67	770.83 ± 48.22	151.50 ± 1.28	8.48 ± 0.67	64.00 ± 0.56	19.88 ± 0.16	310.67 ± 1.80*
HD (2000 mg/kg)	7.56 ± 0.17	9.23 ± 1.06	613.83 ± 34.22	151.83 ± 2.26	9.23 ± 1.06	64.67 ± 0.92	20.17 ± 0.31	308.00 ± 0.73

RBC: red blood cells; WBC: white blood cells; PLT: platelets; HGB: hemoglobin; HCT: hematocrit; MCV: mean corpuscular volume; MCH: mean corpuscular hemoglobin; MCHC: mean corpuscular hemoglobin concentration; HD: high dose; MD: medium dose; LD: low dose; NC: normal control. The treated groups received different doses of crude extract of khat except NC which received distilled water. The values were expressed as mean ± S.E.M. The superscript (*) indicated a significant difference, *P < *0.05.

**Table 7:** Blood film profile of female SD rats.

Dose (mg/kg)	RBC (×10^12^/L)	WBC (×10^9^/L)	PLT (×10^9^/L)	HGB (g/L)	HCT (L/L)	MCV (fl)	MCH (pg)	MCHC (g/L)
NC (10 mL/kg)	8.24 ± 0.14	7.10 ± 0.88	616.33 ± 52.67	155.00 ± 2.35	0.51 ± 0.009	61.167 ± 0.48	18.58 ± 0.15	304.00 ± 1.37
LD (500 mg/kg)	8.22 ± 0.21	8.10 ± 0.37	727.67 ± 43.90	157.67 ± 2.91	0.51 ± 0.011	62.83 ± 0.54	19.35 ± 0.21^a∗^	308.33 ± 1.78
MD (1000 mg/kg)	7.82 ± 0.21	7.95 ± 0.16	760.67 ± 16.41	161.17 ± 2.74	0.50 ± 0.009	62.67 ± 0.49	19.65 ± 0.21^b∗^	315.67 ± 0.49^b∗^
HD (2000 mg/kg)	7.65 ± 0.09	9.80 ± 0.67^a∗^	722.67 ± 51.85	152.50 ± 1.38	0.49 ± 0.005	63.67 ± 0.42^b∗^	19.97 ± 0.21^b∗^	315.67 ± 2.03^b∗^

RBC: red blood cells; WBC: white blood cells; PLT: platelets; HGB: hemoglobin; HCT: hematocrit; MCV: mean corpuscular volume; MCH: mean corpuscular hemoglobin; MCHC: mean corpuscular hemoglobin concentration; HD: high dose; MD: medium dose; LD: low dose; NC: normal control. The treated groups received different doses levels except NC, received distilled water. The values were expressed as mean ± S.E.M. The superscript (*) indicated a significant difference, whereas (^a^) and (^b^) denoted a significant difference at *P *< 0.05 and *P *≤ 0.01, respectively.

## References

[1] Cox G, Rampes H (2003). Adverse effects of khat: a review. *Advances in Psychiatric Treatment*.

[2] Al-Motarreb A, Al-Habori M, Broadley KJ (2010). Khat chewing, cardiovascular diseases and other internal medical problems: the current situation and directions for future research. *Journal of Ethnopharmacology*.

[3] Al-Habori M (2005). The potential adverse effects of habitual use of *Catha edulis* (khat). *Expert Opinion on Drug Safety*.

[4] Kassim S, Islam S, Croucher R (2010). Validity and reliability of a Severity of Dependence Scale for khat (SDS-khat). *Journal of Ethnopharmacology*.

[5] Hoffman R, Al’Absi M (2010). Khat use and neurobehavioral functions: suggestions for future studies. *Journal of Ethnopharmacology*.

[6] Getahun W, Gedif T, Tesfaye F (2010). Regular Khat (*Catha edulis*) chewing is associated with elevated diastolic blood pressure among adults in Butajira, Ethiopia: a comparative study. *BMC Public Health*.

[7] Ali WM, Zubaid M, Al-Motarreb A (2010). Association of khat chewing with increased risk of stroke and death in patients presenting with acute coronary syndrome. *Mayo Clinic Proceedings*.

[8] Nencini P, Ahmed AM (1989). Khat consumption: a pharmacological review. *Drug and Alcohol Dependence*.

[9] Manghi RA, Broers B, Khan R, Benguettat D, Khazaal Y, Zullino DF (2009). Khat use: lifestyle or addiction?. *Journal of Psychoactive Drugs*.

[10] Luqman W, Danowski TS (1976). The use of khat (*Catha edulis*) in Yemen. Social and medical observations. *Annals of Internal Medicine*.

[11] Chapman MH, Kajihara M, Borges G (2010). Severe, acute liver injury and khat leaves. *New England Journal of Medicine*.

[12] Ardouin C, Carteron B, Morvan D (1979). Value of liver needle-biopsy in Djibouti (1,110 specimens). *Medecine Tropicale*.

[13] Coton T, Simon F, Oliver M, Kraemer P (2011). Hepatotoxicity of khat chewing. *Liver International*.

[14] Al-Mamary M, Al-Habori M, Al-Aghbari AM, Baker MM (2002). Investigation into the toxicological effects of *Catha edulis* leaves: a short term study in animals. *Phytotherapy Research*.

[15] Al-Habori M, Al-Aghbari A, Al-Mamary M, Baker M (2002). Toxicological evaluation of *Catha edulis* leaves: a long term feeding experiment in animals. *Journal of Ethnopharmacology*.

[16] Kalix P (1984). The pharmacology of khat. *General Pharmacology*.

[17] Peevers CG, Moorghen M, Collins PL, Gordon FH, M CA (2010). Liver disease and cirrhosis because of Khat chewing in UK Somali men: a case series. *Liver International*.

[18] Stuyt RJL, Willems SM, Wagtmans MJ, Van Hoek B (2011). Chewing khat and chronic liver disease. *Liver International*.

[19] OECD http://www.oecd-ilibrary.org/environment/oecd-principles-on-good-laboratory-practice_9789264078536-en.

[20] OECD http://www.oecd-ilibrary.org/environment/test-no-407-repeated-dose-28-day-oral-toxicity-study-in-rodents_9789264070684-en.

[21] Halbach H (1972). Medical aspects of the chewing of khat leaves.. *Bulletin of the World Health Organization*.

[22] Sireeratawong S, Lertprasertsuke N, Srisawat U (2008). Acute and subchronic toxicity study of the water extract from root of Sida rhombifolia Linn. in rats. *Songklanakarin Journal of Science and Technology*.

[23] Aziz HA, Peh KK, Tan YTF (2011). Herbal delivery system for treatment of obesity administration of encapsulated khat-extracts on body weight of rats. *Obesity Research and Clinical Practice*.

[24] Admassie E, Engidawork E (2011). Subchronic administration of *Catha edulis* F. (khat) extract is marked by elevation of cardiac biomarkers and subendocardial necrosis besides blood pressure alteration in rats. *Journal of Ethnopharmacology*.

[25] Mahmood SA, Lindequist U (2008). A pilot study on the effect of *Catha edulis* frosk.,(celastraceae) on metabolic syndrome in WOKW rats. *African Journal of Traditional, Complementary and Alternative Medicines*.

[26] Al-Mamary M, Molham AH, Abdulwali AA, Al-Obeidi A (2001). In vivo effects of dietary sorghum tannins on rabbit digestive enzymes and mineral absorption. *Nutrition Research*.

[27] Bongard S, Al’Absi M, Khalil NS, Al Habori M (2011). Khat use and trait anger: effects on affect regulation during an acute stressful challenge. *European Addiction Research*.

[28] Nagayasu S, Suzuki S, Yamashita A (2012). Smoking and adipose tissue inflammation suppress leptin expression in Japanese obese males: potential mechanism of resistance to weight loss among Japanese obese smokers. *Tobacco Induced Diseases*.

[29] Ghani NA, Eriksson M, Kristiansson B, Qirbi A (1987). The influence of khat-chewing on birth-weight in full-term infants. *Social Science and Medicine*.

[30] Michalopoulos GK (2007). Liver regeneration. *Journal of Cellular Physiology*.

[31] Ashafa AOT, Yakubu MT, Grierson DS, Afolayan AJ (2009). Toxicological evaluation of the aqueous extract of *Felicia muricata* Thunb. leaves in Wistar rats. *African Journal of Biotechnology*.

[32] Burke MD (2002). Liver function: test selection and interpretation of results. *Clinics in Laboratory Medicine*.

[33] Woodman DD, Evans GO (1996). Assessment of hepatotoxicity. *Animal Clinical Chemistry*.

[34] Pratt DS, Kaplan MM (2000). Evaluation of abnormal liver-enzyme results in asymptomatic patients. *New England Journal of Medicine*.

[35] Ozer J, Ratner M, Shaw M, Bailey W, Schomaker S (2008). The current state of serum biomarkers of hepatotoxicity. *Toxicology*.

[36] Navarro VJ, Senior JR (2006). Drug-related hepatotoxicity. *New England Journal of Medicine*.

[37] Johnston DE (1999). Special considerations in interpreting liver function tests. *American Family Physician*.

[38] Limdi JK, Hyde GM (2003). Evaluation of abnormal liver function tests. *Postgraduate Medical Journal*.

[39] O’Brien PJ, Slaughter MR, Polley SR, Kramer K (2002). Advantages of glutamate dehydrogenase as a blood biomarker of acute hepatic injury in rats. *Laboratory Animals*.

[40] Yegneswaran B, Pitchumoni CS (2010). Q: when should serum amylase and lipase levels be repeated in a patient with acute pancreatitis?. *Cleveland Clinic Journal of Medicine*.

[41] Pratt DS, Kaplan MM (2000). Evaluation of abnormal liver-enzyme results in asymptomatic patients. *New England Journal of Medicine*.

[42] Giannini EG, Testa R, Savarino V (2005). Liver enzyme alteration: a guide for clinicians. *Canadian Medical Association Journal*.

[43] Al-Mamary M, Al-Habori M, Al-Aghbari AM, Baker MM (2002). Investigation into the toxicological effects of *Catha edulis* leaves: a short term study in animals. *Phytotherapy Research*.

[44] Palmes D, Spiegel HU (2004). Animal models of liver regeneration. *Biomaterials*.

[45] Corkery JM, Schifano F, Oyefeso A (2011). Overview of literature and information on “khat-related” mortality: a call for recognition of the issue and further research. *Annali dell'Istituto Superiore di Sanità*.

[46] Al-Akwa AA, Shaher M, Al-Akwa S, Aleryani SL (2009). Free radicals are present in human serum of *Catha edulis* Forsk (Khat) abusers. *Journal of Ethnopharmacology*.

[47] Waner T, Nyska A (1991). The toxicological significance of decreased activities of blood alanine and aspartate aminotransferase. *Veterinary Research Communications*.

